# Role of Csdc2 in Regulating Secondary Hair Follicle Growth in Cashmere Goats

**DOI:** 10.3390/ijms25158349

**Published:** 2024-07-30

**Authors:** Heqing Zhu, Yingying Li, He Xu, Yuehui Ma, Göran Andersson, Erik Bongcam-Rudloff, Tiantian Li, Jie Zhang, Yan Li, Jilong Han, Min Yang

**Affiliations:** 1 College of Animal Science and Technology, Shihezi University, Shihezi 832061, China; 2 State Key Laboratory of Animal Biotech Breeding, Institute of Animal Sciences, Chinese Academy of Agricultural Sciences, Beijing 100193, China; 3 Department of Animal Biosciences, Swedish University of Agricultural Sciences, 75007 Uppsala, Sweden

**Keywords:** Cashmere goat, Csdc2, hair follicle, ChIP-Seq, Robo2

## Abstract

Cashmere goats possess two types of hair follicles, with the secondary hair follicles producing valuable cashmere fiber used for textiles. The growth of cashmere exhibits a seasonal pattern arising from photoperiod change. Transcription factors play crucial roles during this process. The transcription factor, cold-shock domain, containing C2 (Csdc2) plays a crucial role in modulating cell proliferation and differentiation. Our preceding research indicated that the expression of Csdc2 changes periodically during anagen to telogen. However, the mechanisms of Csdc2 in regulating SHF growth remain unclear. Here, we found that the knockdown of Csdc2 inhibits the proliferation of dermal papilla cells. ChIP-Seq analysis showed that Csdc2 had a unique DNA binding motif in SHFs. Through conjoint analysis of ChIP-Seq and RNA-Seq, we revealed a total of 25 candidate target genes of Csdc2. Notably, we discovered a putative Csdc2 binding site within roundabout guidance receptor 2 (*Robo2*) on chromosome 1 of the goat genome. Furthermore, qRT-PCR and dual-luciferase reporter assay confirmed Csdc2’s positive regulatory influence on *Robo2*. These findings expand the research field of hair follicle transcriptional regulatory networks, offering insights into molecular breeding strategies to enhance cashmere production in goats.

## 1. Introduction

Cashmere goats are an economically important livestock species that possess two types of wool, wherein the long hair from primary hair follicles (PHFs) serves for protection, and the undercoat of cashmere produced by secondary hair follicles (SHFs) provides warmth. Cashmere is a highly prized textile material with remarkable economic value, consequently, increasing the yield of cashmere has always been a pivotal goal in breeding research [1,2]. Unlike PHFs, the growth of cashmere from SHFs is periodic and undergoes an annual cycle of cell proliferation (anagen), apoptosis (catagen) and relative mitotic quiescence (telogen) [3]. During hair follicle reconstruction, dermal papilla cells (DPCs) serve as the central growth control center, possessing inductive properties crucial for morphogenesis, development, and hair formation [4]. The periodic growth of SHFs involves changes in hormones, photoperiodicity, and regulatory factors that are essential for comprehending the underlying mechanisms [5]. Various signaling pathways have been identified as influential to hair follicle (HF) development, including Fgf signaling during the telogen-to-anagen transition [6], Tgf-β1 and Tgf-β2 induction of SHFs into catagen [7], variations in Bmp2 expression from late telogen to anagen [8], and Wnt/β-catenin signaling, which regulates cell proliferation in the hair matrix and dermal papilla [9]. Although several signaling pathways have been shown to influence HF development, there is still a need to explore the regulatory network controlling the periodic SHFs.

Studies have shown that transcription factors (TFs) are also involved in the regulation of HF development. TFs are crucial in regulating gene expression by binding to DNA in a sequence-specific manner. TF binding sites can be identified using motifs, representing sequences preferred by a given TF [10]. Foxn1 has been identified as playing an important role in controlling HF keratinocyte differentiation [11]. Similarly, NFIB and its target Edn2 regulate the activation of hair follicle stem cells [12]. Lymphatic enhancer factor 1 (Lef1) is a transcription factor that positively regulates the Wnt/β-catenin signaling pathway. The gene downstream, distal-less homeobox 3 (Dlx3), shares similar functions with Lef1, regulating HF development by promoting the proliferation of primary and secondary DPCs and inhibiting apoptosis [13]. In our previous research, Csdc2 was identified as a potential TF involved in regulating the cyclical processes of SHFs in Cashmere goat skin tissues [14]. Csdc2 is an unstable hydrophilic protein residing in the cell nucleus and functions within the cytoplasm, it is classified as an RNA-binding protein (RBP) featuring a cold-shock domain (CSD) with RNP1 and RNP2 motifs and is capable of binding to DNA and double-stranded RNA during post-transcriptional processes [15,16,17]. The expression of Csdc2, regulated by temperature, is found in the heart, ovaries, testes, skeletal muscle, and nervous system and plays a significant role in neuronal development and maturation [18], decidualization of the endometrium, and cellular proliferation [19,20]. However, the molecular mechanisms of Csdc2 in regulating SHF growth remain unclear.

Our previous research found that Csdc2 is highly expressed during anagen (August to December) and downregulated during telogen (February to April). Knockdown of *Csdc2* in mouse 3T3 cells led to reduced expression of HF-related genes and decreased cell proliferation rates, suggesting that Csdc2 is important in regulating the cyclical growth of SHFs [14]. To further explore the function and regulatory mechanism, we utilized chromatin immunoprecipitation followed by sequencing (ChIP-seq) and dual-luciferase reporter assay (DLR) to identify potential target genes. Here, we demonstrated that Csdc2 is a positive regulator of hair follicle growth and identified roundabout guidance receptor member 2 (*Robo2*) as a potential target gene of Csdc2. This research sheds light on the regulatory mechanisms of SHF growth in Cashmere goats.

## 2. Results

### 2.1. Immunohistochemical Analysis of SHFs

**Figure 1:**
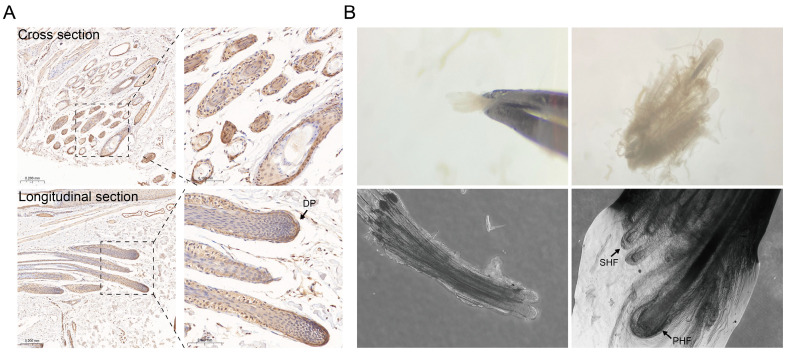
Immunohistochemistry and two types of HFs under a stereomicroscope. (**A**) Immunohistochemical results show Csdc2 is highly expressed at DP in anagen (Sep). Scale bars indicate 100 µm. (**B**) PHFs and SHFs.

All immunohistochemistry (IHC) slices prepared from anagen phase skin were observed by light microscope (Olympus, Shinjuku, Japan). IHC revealed that Csdc2 was predominantly localized in the secondary hair papilla in September (Figure 1A). Clusters of SHFs surrounding a PHF were observable under a stereomicroscope (Figure 1B). To further explore the regulatory mechanism, we cultured DPCs and carefully isolated the hair bulb from the SHFs during the anagen phase using microsurgical tweezers in preparation for the ChIP experiment.

### 2.2. Characterization of DPCs

**Figure 2:**
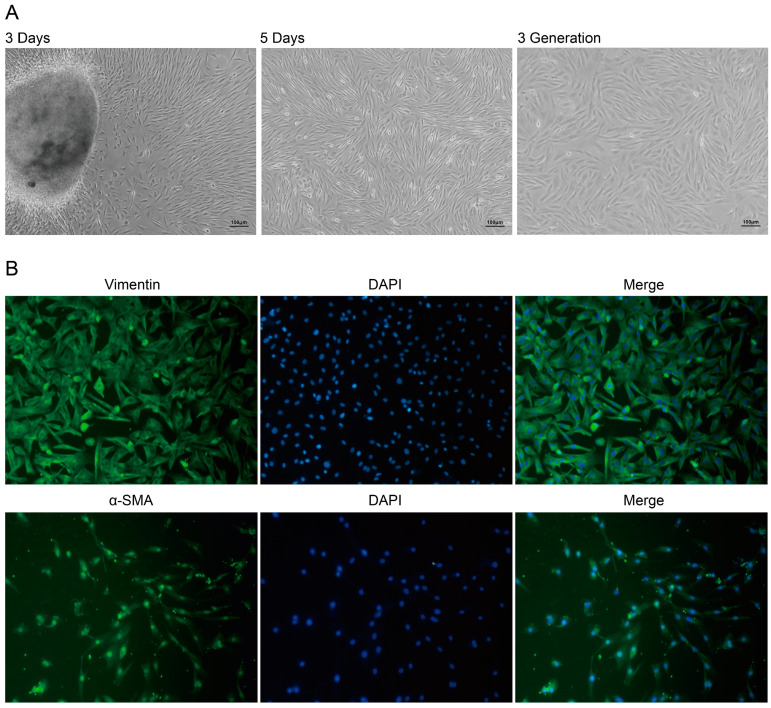
Culture and characterization of DPCs. (**A**) Different states of DPCs at different days of culture. Scale bars indicate 100 µm. (**B**) Using immunofluorescence showing DPCs were positive for vimentin and α-SMA.

DPCs, as a subset of dermal fibroblasts, were characterized by their spindle-like morphology and specific expression of vimentin and α-smooth muscle actin (α-SMA). Cells isolated from fetal cashmere-goat skin-tissue blocks grew rapidly and uniformly, reaching a density of 80% after 5 days in culture (Figure 2A). As expected, immunofluorescence confirmed that the cells were positive for the expression markers vimentin andα-SMA (Figure 2B).

### 2.3. Signaling Pathway Genes Associated with Csdc2 in DPCs

**Figure 3:**
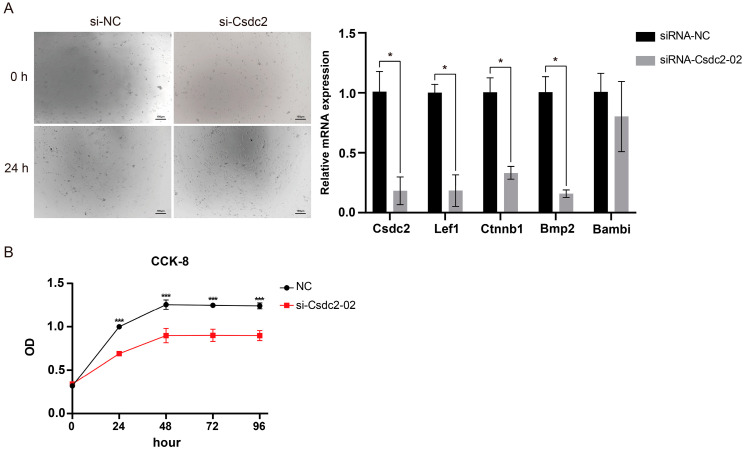
Alterations of hair follicle development-related genes and cell proliferation after Csdc2 knockdown. (**A**) Knockdown of Csdc2 significantly inhibited the expression of HF-development-related genes. (**B**) Cell proliferation after Csdc2 knockdown. *** *p* < 0.001, * *p* < 0.05.

Following interference pre-experiment screening, si-Csdc2-02 showed the highest interference efficiency (Supplementary File S1: Figure S1) and was selected for subsequent knockdown experiments. To identify the effect of Csdc2 on key signaling pathways that have been identified as influential to hair follicle (HF) development, the qRT-PCR results showed that knockdown of *Csdc2* significantly decreased the expression of key Wnt signaling pathway proteins Lef1 and β-catenin (*p <* 0.05). Additionally, the expression of Tgf-β superfamily members Bmp2 (*p <* 0.05) and Bambi was also reduced (Figure 3A). Next, we analyzed the effect of knockdown of *Csdc2* on the proliferation of dermal fibroblasts. The results of the cell proliferation experiment suggested a significant inhibition of cell proliferation 24 h after knocking down Csdc2 compared to the control group (*p <* 0.001, Figure 3B). These results indicated that Csdc2 plays a significant role in regulating the transcription of these genes involved in HF development.

### 2.4. Identification of the Genome-Wide Csdc2 Binding Sites in HF

**Figure 4:**
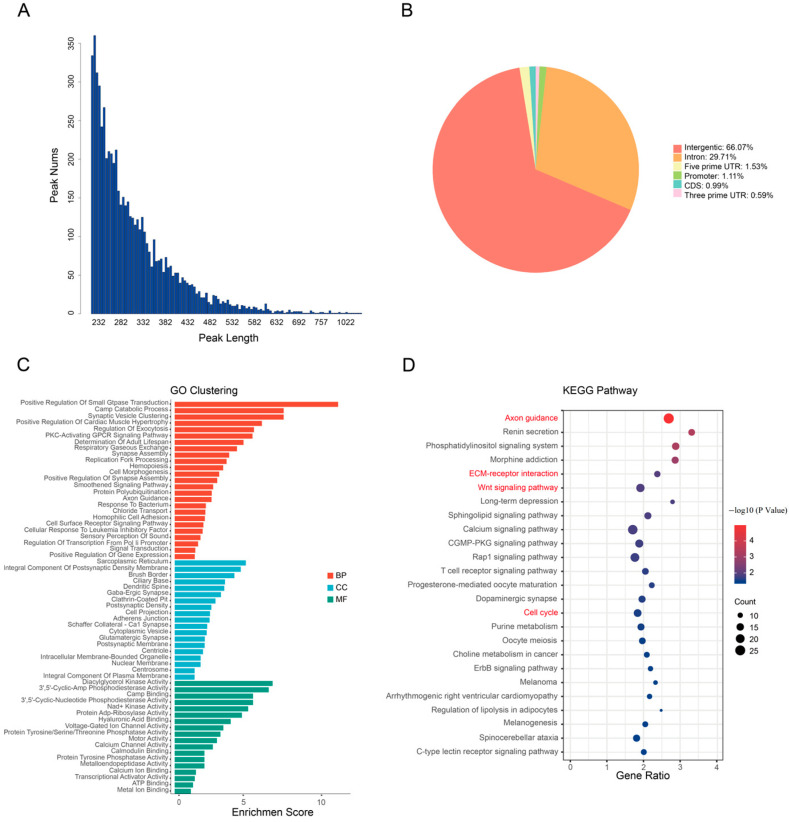
Analysis results of ChIP. (**A**) Average length of annotated peaks. (**B**) Regional statistics of peak annotation. (**C**) GO cluster of genes in coding and non-coding regions. (**D**) KEGG analysis of genes in coding and non-coding regions.

The raw data from the Csdc2 ChIP experiment and the control group (Csdc2 Input) were 5.692 billion bp and 6.223 billion bp, respectively. After initial processing, 93.48% of the Csdc2 ChIP data and 97.04% of the Csdc2 input data were retained for analysis. The quality of the filtered sequences was high, with Q-scores exceeding 30, indicating reliable sequencing results. Alignment with the reference genome yielded mapping rates of 95% for the ChIP data and 98% for the input data, providing a solid foundation for subsequent analyses (Supplementary File S2: Table S1). Peak calling identified 6117 peaks, with an average length of 321.58 bp. Most of the peaks were within the 200–300 bp range (Figure 4A). Annotation of the 6117 identified peaks within the goat genome revealed comprehensive location details: 66.07% were in intergenic regions and 33.93% were within gene coding and non-coding regions, including 2 kb upstream and downstream of transcription start sites (TSS). Specifically, introns contained 29% of peaks, promoters 1.1%, coding sequences (CDS) 0.99%, 5′ UTR 1.53%, and 3′ UTR 0.59% (Figure 4B). After removing peaks annotated as intergenic regions, 2063 peaks were retained. GO analysis revealed significant enrichment for genes associated with these peaks: 25 were in biological processes (BPs), 19 were in cellular components (CCs), and 18 were in molecular functions (MFs). These genes are primarily involved in various biological processes, such as cell proliferation, signal transduction, and feedback regulation. Notably, they exhibit enrichment in cellular components like cell membrane structures, organelles, and intracellular proteins. Additionally, they are predominantly associated with molecular functions encompassing DNA binding, transcriptional activity, and molecular structure (Figure 4C). Moreover, 25 significant signaling pathways were identified, including the Wnt signaling pathway, ECM-receptor interaction, cell cycle, and axon guidance, all of which are pertinent to HF growth and development (Figure 4D).

### 2.5. Motif Analysis of Csdc2 Binding Sites

**Figure 5:**
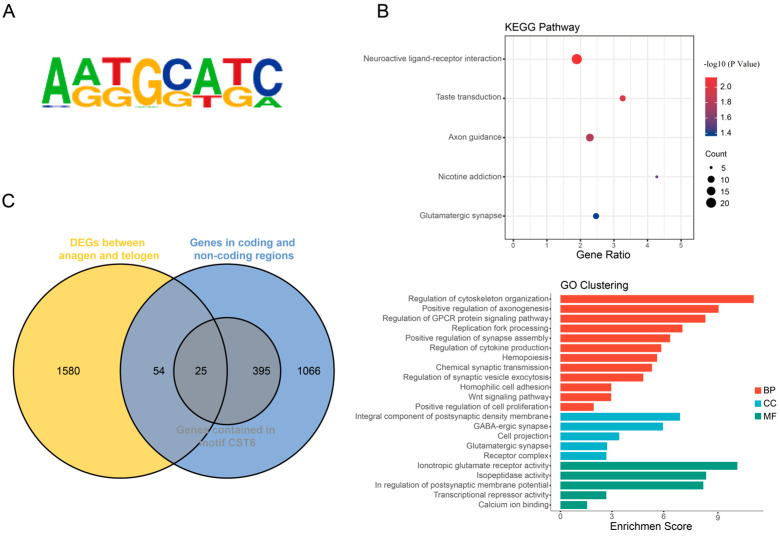
Specific binding sites analysis. (**A**) Sequence map of motif CST6. (**B**) KEGG enrichment and GO clustering of genes in this motif. (**C**) Venn map shows the relationship between ChIP data, RNA-Seq data, and motif corresponding genes.

Transcription factor binding sites identified in ChIP-Seq often contain short, recurring motifs with characteristic patterns. Csdc2 comprises a CSD that can recognize motifs with high GA content at their binding sites, and the target sites of the CSD contain the GGAG sequence [21,22]. In the motif prediction results generated using HOMER, we identified a motif CST6, which contains a GGAG sequence (Figure 5A) and is associated with the highest number of peaks among all similar results in both coding and non-coding regions (Supplementary File S2: Table S2; Supplementary File S3: Data S1). GO analysis revealed that the genes are predominantly involved in biological processes related to cell proliferation and signaling, including axon guidance, cytoskeleton regulation, growth factor binding, and transcriptional repression. KEGG enrichment analysis identified three pathways associated with HF development—axon guidance, neuroactive ligand-receptor interaction, and glutamatergic synapse—suggesting that this motif may be a potential binding site for Csdc2 (Figure 5B).

### 2.6. Joint ChIP-Seq and RNA-Seq Analysis to Screen Target Genes

**Figure 6:**
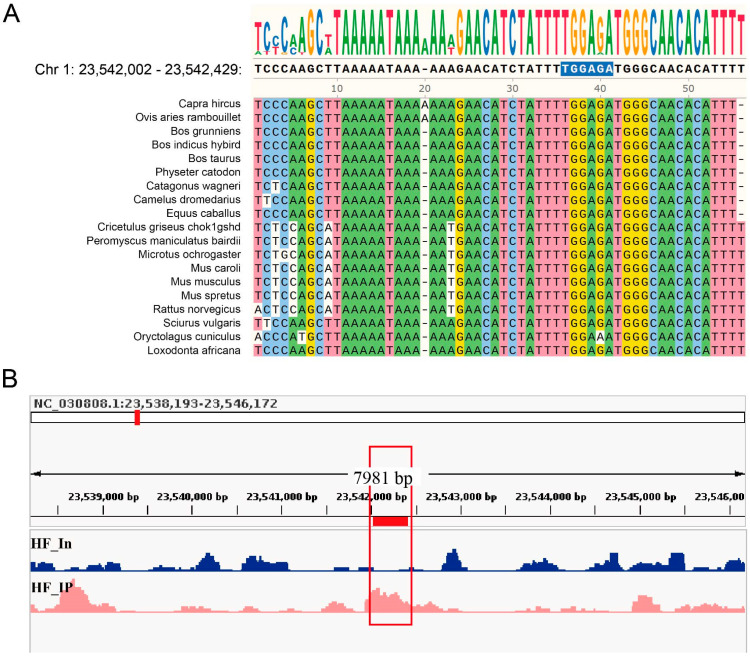
Analyses of sequence conservation and visualisation of binding sites. (**A**) Conservation of motifs. (**B**) Enrichment of Csdc2 in the intron 6 of *Robo2*.

Our previous studies have shown that Csdc2 exhibits significant differences at different phases of HF development [14]. To identify target genes that may co-regulate HF development with Csdc2, we combined 1540 genes from ChIP peaks (Supplementary File S3: Data S2) with 1660 genes that had significant differential expression in HFs during the pre-anagen to anagen and the catagen to telogen transitions as identified by RNA-Seq analysis in our studies (Figure 5C). This integrated analysis yielded a total of 79 genes, and further analysis with target motif CST6 enriched genes narrowed down to 25 common genes (Supplementary File S3: Data S3). Conservation analysis across multiple mammal species revealed a highly conserved sequence (AAAGAACATCTATTTTGGAGATGGGCAACACAT) within the peak region annotated as roundabout guidance receptor 2, containing sequence TGGAGA located at Chr 1: 23,542,002–23,542,429 that motif CST6 enriched, likely a binding site for Csdc2 (Figure 6A). Visualization analysis of the peak calling revealed the binding of Csdc2 to this site (Figure 6B). Based on these findings, we hypothesize that *Robo2* could be a target gene of Csdc2.

### 2.7. Regulatory Relationships and Expression Patterns of Target Genes

**Figure 7:**
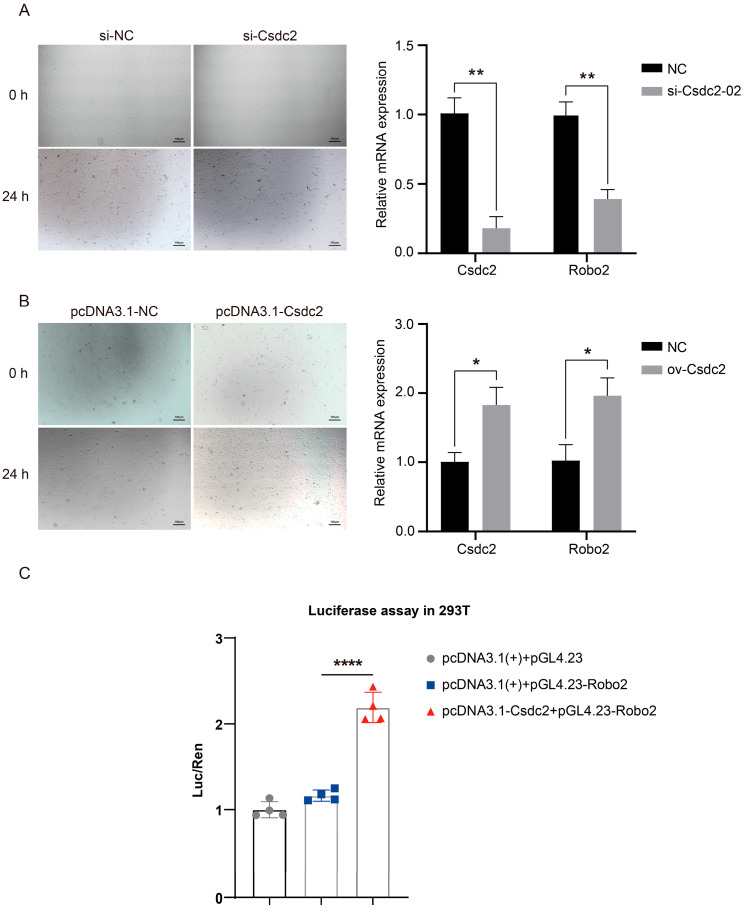
Knockdown, overexpression, and dual luciferase reporter assay results. (**A**) Overexpression Csdc2 promoted the expression of Robo2. (**B**) Knockdown of Csdc2 significantly inhibited the expression of Robo2. (**C**) The results of the luciferase reporter assays conducted in 293T cells indicate a targeting relationship between Robo2 and Csdc2 (mean ± SD). *, *p* < 0.05; **, *p* < 0.01, ****, *p* < 0.0001.

Confirmation through effective Csdc2 interference using siRNA-Csdc2 and overexpression assays highlighted that si-Csdc2 transfection significantly reduced the mRNA levels of both Csdc2 (*p <* 0.05) and Robo2 (*p <* 0.05), demonstrating that *Csdc2* interference suppresses *Robo2* expression (Figure 7A). Conversely, 48 h post-transfection with the pcDNA3.1 (+)-Csdc2 overexpression vector, there was a notable increase in the mRNA levels of *Csdc2* and *Robo2* (*p <* 0.05), indicating that *Csdc2* overexpression enhances the expression of *Robo2* (Figure 7B). Moreover, the fluorescence intensity of the Csdc2 + Robo2 group was significantly higher than that of the pcDNA3.1 (+)+Robo2 group (*p <* 0.0001), indicating that *Csdc2* enhances the expression of *Robo2*. These results point towards a targeted regulatory relationship between *Csdc2* and *Robo2* (Figure 7C).

## 3. Discussion

Cashmere goats are highly prized for their ability to produce cashmere, a luxurious natural fiber known for its outstanding textile qualities and considerable economic value. The production of cashmere is contingent upon the growth and regeneration of SHFs, which undergo cyclical developmental phases known as anagen, catagen, and telogen [23,24]. This process is influenced by several genetic factors, such as Wnt signaling pathways, Tgf-β pathways, and Bmps, as well as hormones like melatonin and prolactin. Previous studies have demonstrated the transcription factor Csdc2’s role in regulating proliferation across various cell types, including brain tumor cells and endometrial stromal cells [18,19,20]. Our research has shown that Csdc2 is also involved in the development of SHFs; however, the specific downstream genes that interact with Csdc2 remain unknown. In this study, we first demonstrated that Csdc2 knockdown affects cellular proliferation and the expression of signaling pathway genes related to SHF growth. Moreover, we validated through ChIP assays, dual luciferase assays, and qRT-PCR that Csdc2 positively regulates *Robo2* by binding to the introns of the target gene *Robo2*. Our findings lay the groundwork for further elucidating the mechanisms of SHF growth.

Firstly, we explored the expression of Csdc2 in SHFs during the anagen phase via immunohistochemistry and discovered that Csdc2 is primarily expressed in the hair bulb, suggesting a critical role in regulating SHF growth. To delve further into the mechanism, we employed a gene knockdown strategy to evaluate the effect of *Csdc2* on the proliferation of DPCs and the expression of key genes associated with HF development and cyclic growth. Knockdown of *Csdc2* resulted in inhibited cell proliferation (*p <* 0.001) and a decrease in the mRNA levels of *Lef1*, *Ctnnb1*, and *Bmp2* (*p <* 0.05), which are genes crucial for HF development and cyclic growth. The Wnt/β-catenin signaling pathway is a key pathway for the transition of HFs from telogen to anagen. Lef1 and β-catenin form a dimer complex, regulating the transcription of downstream target genes and affecting the proliferation, migration, and adhesion of HF cells [25]. The Bmp signaling pathway is considered an inhibitor of HF development [26], while Bambi is a negative regulator of the Bmp2 signaling pathway [27]. Bmp2 is gradually expressed in pre-anagen, reaches a peak, and then gradually decreases, reaching its lowest point in late telogen [28]. During an HF cycle, Bmp2 gradually accumulates and exceeds proliferative signals such as Wnt and Egf, inducing HFs to enter catagen [29]. In this study, the knockdown of Csdc2 led to reduced expression levels of these genes, indicating that Csdc2 plays a significant role in regulating SHF growth.

Next, we utilized ChIP-seq analysis to identify genes regulated by Csdc2. Analysis of the sequencing data revealed a total of 6117 peaks corresponding to the binding sites of Csdc2 after aligning with the goat genome. Of these peaks, 4.1% were in the transcription start site (TSS) regions, with the majority found in intergenic and intron. TFs also have the capacity to interact with regions beyond promoters, influencing gene expression and showing a greater propensity to bind to both gene-coding and non-coding regions [30,31]. For instance, alterations in the expression levels of specific miRNAs have been shown to regulate the activity of heat-shock proteins [32]. Consequently, we removed peaks located in intergenic regions and concentrated on the remaining peaks associated with coding and non-coding regions. GO and KEGG enrichment analyses were then carried out. The analysis indicated that genes associated with these peaks are primarily involved in pathways related to cell proliferation, migration, and HF development. We subsequently compared the genes linked to these peaks with the differentially expressed genes (DEGs) in HFs during various phases, as identified in our previous study [14]. In total, we identified 79 genes showing differential expression in both datasets.

In eukaryotic organisms, transcription factor binding sites are typically characterized by conserved short sequence motifs ranging from 5 to 15 bp in length [33]. Our study indicates that motif analysis of Csdc2 binding peaks revealed significant enrichment of the CST6 (MacIsaac)/Yeast motif, appearing in 11.65% of the binding peaks. Subsequent GO and KEGG analyses indicated that genes annotated with this motif are enriched in signal transduction pathways associated with HF development, such as axon guidance, receptor interaction, and transcriptional inhibition. Cells rely on specific signals to direct their growth during development, like axon guidance in neuronal regeneration [34]. Receptors like Fgf7 influence hair growth by regulating cell growth and differentiation through the MAPK pathway activation via Fgfr2 binding [35]. In the cyclic development of HFs, transcriptional repressors timely activate to inhibit downstream gene transcription. For instance, the transient expression of Foxc1 maintains stem cell adhesion, promoting the transition of HFs into telogen [36]. KEGG and GO annotations for enriched peaks reveal a strong link between motif CST6 and HF development. Subsequent joint analysis with RNA-Seq data refined the list of candidate genes to 25. Studies have indicated that transcription factor binding sites exhibit higher evolutionary conservation [37], and our analysis has revealed that *Robo2* contains a potential CST6 motif, TGGAGA, which is highly conserved among 18 mammalian species, suggesting it is a candidate target gene of Csdc2. Roundabout guidance receptors (Robo) possess immunoglobulin-like (Ig) domains that facilitate intercellular interactions within the nervous system. Of the four recognized mammalian Robo subtypes (Robo1–4), Robo2’s extracellular domain comprises five Ig and three fibronectin type III (FNIII) domains [38,39]. Robo2 plays an important physiological role in cell proliferation and migration, developmental processes of the nervous, cardiovascular, and urinary systems [40,41], as well as methylation of melanoma sites [42]. Recent studies have underscored the significance of Robo2 in nerve and soft tissue development [43,44], but its role in HF development has not yet been investigated. The bioinformatics analysis results suggested that *Robo2* may be the target gene of Csdc2, which needs to be verified.

The cellular assays confirmed that *Robo2* mRNA expression in DPCs was significantly reduced 24 h post-transfection with *Csdc2* siRNA (*p <* 0.01). Meanwhile, *Csdc2* overexpression markedly enhanced *Robo2* expression levels (*p <* 0.05), indicating a potential regulatory interaction between *Csdc2* and *Robo2* at the mRNA level. The fluorescence intensity in cells co-transfected with both plasmids was significantly higher than in the control group (*p <* 0.0001), as determined by DLR. In summary, the analysis of ChIP-Seq data, RNA interference of *Csdc2*, overexpression, and dual-luciferase assays all support the targeting relationship between Robo2 and Csdc2. Our findings suggest that Csdc2 can impact the expression of key genes in the Wnt/β-catenin signaling pathway, establishing the groundwork for further study on how Csdc2 and Robo2 regulate the periodic growth of HFs.

## 4. Materials and Methods

### 4.1. Experimental Materials

Five two-year-old healthy female Cashmere goats with similar body weights were raised at the Animal Science and Technology College Experimental Station of Shihezi University in Shihezi, Xinjiang, China. These goats were raised in a shared environment with unrestricted access to food and water. After depilation of the scapular hair and administration of local anesthesia to each goat, a 1 cm^²^ skin sample was taken using sterile ophthalmic scissors from the scapular. Half of the samples were fixed in 4% paraformaldehyde for IHC and the other half were placed in 1× PBS containing 1% penicillin-streptomycin for follicle collection. Yunnan Baiyao hemostatic powder was evenly applied to the skin wound of the goats. Skin samples of three 70-day fetal-age Cashmere goats, used for the isolation and culture of DPCs, were obtained from the municipal slaughterhouse in Shihezi that complies with China’s ‘Regulations on the Administration of Livestock and Poultry Slaughter’. The pre-slaughter cashmere goats were sourced from non-epidemic areas with animal quarantine certifications. All animal experiments were authorized by the Biology Ethics Committee of Shihezi University. The ethics committee approval number is A2020-34.

### 4.2. Immunohistochemistry

Skin tissues from cashmere goats were fixed overnight in 4% PFA. After three washes with distilled water, the tissues were embedded in paraffin with a melting point of 56 °C. Tissue sections (5 µm thick) were cut using a microtome (Leica Biosystems, Wetzlar, Germany) and mounted on glass slides. Antigen retrieval was performed using heat-induced epitope retrieval (HIER) with 10 mM citrate buffer at pH 6.0. Each slide was incubated with 3% H_2_O_2_ for 10 min at room temperature, then washed with PBS for 5 min and blocked with serum. The slides were then incubated with primary antibody (rabbit anti-Csdc2 antibody, dilution 1:200) overnight at 4 °C, followed by secondary antibody (goat anti-rabbit IgG H&L (HRP), dilution 1:200, Cat. no. D110058, Sangon Biotech, Shanghai, China) for 50 min at room temperature. The slides were washed three times with PBS and visualization using DAB staining. 

### 4.3. Dermal Papilla Cells Culture and Characterization

DPCs were cultured from tissue blocks in a complete culture medium (90% DMEM, 10% fetal bovine serum, 1% penicillin-streptomycin) and incubated at 37 °C with 5% CO_2_. When the monolayer cell density of the primary culture reached 80%, the tissue blocks were removed, and the cells were sub-cultured and purified via time differential digestion. Immunofluorescence staining was used to identify the cell types. Following fixation with 4% paraformaldehyde, permeabilization, and blocking with 20% BSA, the cells were incubated with primary antibodies (anti-VIM rabbit monoclonal antibody, 1:200, Cat. no. D291648, Sangon Biotech, Shanghai, China; anti-alpha SMA, 1:200, Cat. no. ab7817, Abcam, Cambridge, UK) overnight at 4 °C. After washing with PBS, the secondary antibody was added. The cells were counterstained with DAPI, and the samples were examined under a fluorescence microscope (ZEISS, Oberkochen, Germany). Mean Fluorescence Intensity (MFI) was quantified using Fiji software (v. 2.14.0).

### 4.4. Small Interfering RNA (siRNA) Synthesis, Plasmid Construction, and Cell Transfection

Three small interfering RNAs (siRNAs) and negative controls (NC) were designed and synthesized (Sangon Biotech, Shanghai, China), and the full-length Csdc2 CDS region of goat complementary DNA was cloned into the pcDNA3.1 (+) vector (Tsingke, Beijing, China) to knockdown or overexpress target genes by cell transfection. DPCs were seeded on the 12-well cell petri dish one day prior, and divided into knockdown groups, overexpression groups, and their respective negative control groups. Each group had three replications. Transfection was performed according to the instructions of *TransIntro*^®^ EL Transfection Reagent (Cat. no. FT201; TransGene, Beijing, China). The siRNA sequences are listed in Supplementary File S2, Table S3.

### 4.5. Cell Proliferation Assay

DPCs were seeded at a density of 2.0 × 10^4^ mL^−1^ in 96-well plates. The knockdown group was transfected with siRNA-Csdc2-02, and the negative control group was transfected with 10% Opti-MEM (Cat. no. 31985070; Gibco, Waltham, MA, USA). Each group had five replications. Cell proliferation rates were assessed using the cell counting kit-8 (CCK-8) (Cat. no. E606335; Sangon Biotech, Shanghai, China) according to the manufacturer’s instructions.

### 4.6. Chromatin Immunoprecipitation (ChIP) Assay

All materials and methods for ChIP-seq experiments have been described [45]. After local anesthesia, a 1 cm^2^ piece of skin was excised from the shoulder of each goat using sterile surgical techniques. Skin strips were digested in a solution of 0.2% collagenase D (Cat. no. M5250; Sigma, St. Louis, MO, USA) at 37 °C for 30 min. SHFs were carefully picked out from the digested strips under a dissecting microscope and placed in 1× PBS. Approximately 500 mg of SHF samples were collected for ChIP experiments. The follicle samples were divided into the ChIP group and the input group (control). Chromatin was isolated using a chromatin immunoprecipitation assay kit (Cat. no. P2078; Beyotime, Shanghai, China), followed by incubation with an anti-Csdc2 antibody (Cat. no. orb32452; Biorbyt, Cambridge, UK) for subsequent ChIP-seq analysis. The resulting ChIP-seq reads were aligned to the goat genome (GCF_001704415.2_ARS1.2, NCBI) using Bowtie2 (v. 2.5.1). Peak calling, genomic annotation of Csdc2 peaks and visualization were performed using MACS2 (v. 2.2.9.1) and IGV (v. 2.16.0). Gene functional element distribution was mapped by ChIPseeker [46], while motif finding and annotation were performed using HOMER (v. 4.11). Gene ontology (GO) enrichment analysis of the identified genes was clustered by molecular function (MF), biological processing (BP), and cellular component (CC) to identify significant functions of genes. Pathways analysis was accomplished through the Kyoto Encyclopedia of Genes and Genomes (KEGG) enrichment analysis. The results with *p*.adjust < 0.05 were screened for significant enrichment.

### 4.7. Dual Luciferase Assay

To validate the relationship between Csdc2 and the candidate target gene *Robo2*, a dual luciferase gene reporter kit (Cat. no. FR201; TransGene, Beijing, China) was used for dual luciferase measurement. ChIP-seq analysis revealed potential Robo2 binding site enrichment within the intron region. A conserved 33 bp sequence from this region was cloned into the pGL4.23 vector to generate the pGL4.23-Robo2 plasmid. Co-transfection of 293T cells was performed with the firefly luciferase reporter plasmid and a Csdc2 overexpression plasmid. An experimental group, a control group, and a blank group transfected with empty vectors were set up. Each group had four replications. The activity of the reporter gene was evaluated using the dual-luciferase reporter assay system, and the fluorescence signals of firefly and Renilla luciferases were captured using a multifunctional fluorescence device (Bio-Tek, Waltham, MA, USA). The recorded fluorescence values for each experimental group were then utilized to evaluate the regulatory effect of Robo2.

### 4.8. RNA Extraction and Quantitative Real-Time Polymerase Chain Reaction (qRT-PCR)

Total RNA was extracted using the *TransZol* Up kit (Cat. no. ET111; TransGene, Beijing, China), and then reverse transcription was performed using a cDNA synthesis kit (Cat. no. AT411; TransGene, Beijing, China). In our study, the housekeeping gene *β-actin* was used as the control gene, quantitative real-time PCR was conducted on a *LightCycler*^®^ 96 real-time PCR system (Roche Diagnostics, Rotkreuz, Switzerland) using ArtiCanATM SYBR qPCR Mix (Cat. no. TSE501; Tsingke, Beijing, China), data analysis was performed using the 2^−ΔΔCT^ method. The primer sequences are shown in Supplementary File S2, Table S4.

### 4.9. Statistical Analysis

In this study, the data are presented as mean (x) ± standard deviation (SD). The student’s *t*-test was used for comparing the means of two groups, while an ANOVA was utilized to compare multiple groups: *, *p <* 0.05; **, *p <* 0.01, ***, *p* < 0.001.

## 5. Conclusions

The results of this study indicated that (1) Csdc2 is primarily expressed in the hair bulb of secondary hair follicles; (2) knocking down Csdc2 results in reduced expression of key genes within signaling pathways related to hair follicle development; and (3) Robo2 functioned as a target gene of Csdc2, which was evidenced by the binding of Csdc2 to the Robo2 intron. Our research provides a theoretical framework for the advanced examination of Csdc2’s function in secondary hair follicle cycling and establishes a groundwork for elucidating the molecular mechanisms underpinning this process in cashmere goats.

## Data Availability

The data that support the findings of this study are available from the corresponding author upon reasonable request.

## Supplementary Materials

The supporting information can be downloaded at: https://www.mdpi.com/article/10.3390/ijms25158349/s1.
