# Supplementary material for: Role of Csdc2 in Regulating Secondary Hair Follicle Growth in Cashmere Goats
Source: Int J Mol Sci. 2024 Jul 30;25(15):8349. doi: 10.3390/ijms25158349 (PMC11313070; doi:10.3390/ijms25158349)
Supplement: Supplementary file 1 [file ijms-25-08349-s001.zip › ijms-3065641-supplementary/Supplementary File/Supplementary File S2.docx]

| Table S1 Sequencing data information | | | | | | | | | | |
| --- | --- | --- | --- | --- | --- | --- | --- | --- | --- | --- |
| Sample | RawReads | | RawBases | CleanReads | CleanBases | CleanRatio | Q20 | Q30 | | GC |
| HF_IP | 37950412 | | 5692561800 | 35477556 | 5176858030 | 93.48% | 97.75% | 93.03% | | 42.95% |
| HF_In | 41486952 | | 6223042800 | 40260516 | 5815822120 | 97.04% | 97.75% | 93.02% | | 43.73% |
| \| Table S2 The Top5 motif in Csdc2 binding peaks \| \| \| \| \| \| --- \| --- \| --- \| --- \| --- \| \| Rank \| % of target \| p value \| Motif \| Best match \| \| 1 \| 11.65% \| 1.00E-04 \| 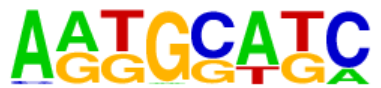 \| CST6 \| \| 2 \| 8.03% \| 1.00E-04 \| 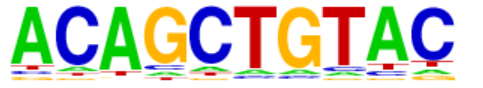 \| Myf5 \| \| 3 \| 4.13% \| 1.00E-10 \| 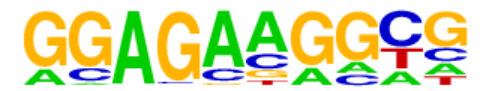 \| Trl(Zf) \| \| 4 \| 4.03% \| 1.00E-08 \| 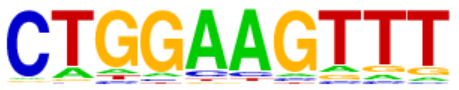 \| GABPA(ETS) \| \| 5 \| 3.16% \| 1.00E-08 \| 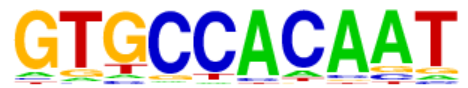 \| MET32 \| | | | | | | | | |  |  |
| Table S3 The sequence of siRNAs used in this study | | | | | | | | |  |  |
| siRNA name | | Sequence(5'-3') | | | | | | |  |  |
| si-*Csdc2*-01 | | AGAACUGCUUACAGACGCCTT | | | | | | |  |  |
| si-*Csdc2*-02 | | AUGUCAGACACGUGCACGATT | | | | | | |  |  |
| si-*Csdc2*-03  si-*Csdc2*-NC | | AACUUCUGGUUCUUGGGCGTT  ACGCGUAUGGAUCGACAAGCC | | | | | | |  |  |

| Table S4 Primer Sequences of RT-qPCR used in this study | | |
| --- | --- | --- |
| Gene | Primer sequences | Product length (bp) |
| *Csdc2* | F：CCGTGTTCAAGGGCGTCTGTAAG | 85 |
|  | R：AAGATGTCCTCCGACCCGTTCTC |  |
| *Robo2* | F：TCCGAGGCGTGGCTTCTTCT | 187 |
|  | R：GGCTGCGGAGGTTGATTATTGC |  |
| *Vimentin* | F：CGTGATGTCCGTCAGCAGTA | 114 |
|  | R：GTCGTTGTTGCGGTTAGCAG |  |
| *Sox2* | F：GATCAGCATGTACCTCCCCG | 116 |
|  | R：GTATGCCGTTAATGGCCGTG |  |
| *VCAN* | F：ATGGGACCGGACAGAACTAC | 156 |
|  | R：GAGTGATTCTGCGGCTGATCT |  |
| *Sox9* | F：CCACCCGGACTACAAGTACCA | 107 |
|  | R：AGATGGCGTTGGGCGAGATG |  |
| *PCNA* | F：CGAGGGCTTCGACACTTACC | 140 |
|  | R：GTGCCAAGGTGTCCGCATTA |  |
| *LEF1* | F: CACACAACTGGCATCCCTCATCC | 121 |
|  | R: GCTCCTGCTCCTTTCTCTGTTCATG |  |
| *CTNNB1* | F: CGGCAATCAAGAAAGCAAGCTCATC | 130 |
|  | R: CACAGACAGCACCTTCAGCACTC |  |
| *β-actin* | F：TCTGGCACCACACCTTCTAC | 102 |
|  | R：TCTTCTCACGGTTGGCCTTG |  |
