# Supplementary figures and images for: Role of Csdc2 in Regulating Secondary Hair Follicle Growth in Cashmere Goats
Source: Int J Mol Sci. 2024 Jul 30;25(15):8349. doi: 10.3390/ijms25158349 (PMC11313070; doi:10.3390/ijms25158349)

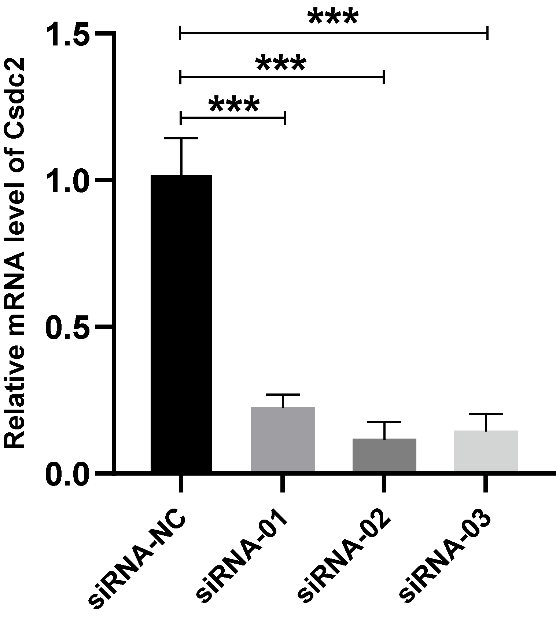
**Figure S1.** Screening of *Csdc2* interference fragments. Data were presented as the mean ± SD, n = 3. ***, *P* < 0.001.

Supplement: Supplementary file 1 [file ijms-25-08349-s001.zip › ijms-3065641-supplementary/Supplementary File/Supplementary File S1.docx]
